# Anchoring of Copper(II)–Schiff Base Complex in SBA-15 Matrix as Efficient Oxidation and Biomimetic Catalyst

**DOI:** 10.3390/ijms25021094

**Published:** 2024-01-16

**Authors:** Mihaela Mureseanu, Irina Bleotu, Cezar-Ionuț Spînu, Nicoleta Cioatera

**Affiliations:** 1 Department of Chemistry, University of Craiova, 107 I Calea Bucureşti, 200478 Craiova, Romania; mmureseanu32@gmail.com (M.M.); spinu_cezar@yahoo.com (C.-I.S.); 2 Faculty of Chemical Engineering and Environmental Protection, Technical University of Iasi, 71 D. Mangeron, 700050 Iasi, Romania; georgi_irina@yahoo.com

**Keywords:** catalyst, SBA-15, Schiff bases, Cu(II) complexes, cyclohexene oxidation, superoxide dismutase activity

## Abstract

A new mononuclear Cu(II) complex [Cu(L_2_)(H_2_O)_2_], where L is the Schiff base 2-[2-(3-bromopropoxy)benzylideneamino] benzoic acid, was synthesized and covalently anchored onto an amino-functionalized SBA-15 mesoporous silica in order to obtain an efficient heterogeneous catalyst. The elemental, structural, textural and morphological characterization confirmed the coordination of the central Cu(II) ion with two ligands and two H_2_O molecules in the synthesized complex and its successful immobilization into the inner pore surface of the NH_2_-functionalized support without the loss of the mesoporous structure. The catalytic activity of the free or immobilized Cu(II) complex was tested in the oxidation of cyclohexene with H_2_O_2_ under an air atmosphere and the dismutation reaction of the superoxide radical anions with very good results. In addition, catalyst reuse tests claim its suitability in alkene oxidation processes or as a biomimetic catalyst.

## 1. Introduction

The selective oxidation of hydrocarbons to oxygen-containing compounds is a significant process in the chemical industry and scientific research for the production of valuable functionalized chemicals [1,2]. In particular, the allylic oxidation of olefins into unsaturated ketones and alcohols is important in organic chemistry and the chemical industry [3,4]. The selective oxidation of cyclohexene is a complex process that can lead to the formation of various products, and the choice of catalyst and reaction conditions plays a crucial role in determining the selectivity of the reaction [3].

Natural enzymes are highly efficient and selective catalysts. Thus, superoxide dismutases (SODs) are a group of natural metalloenzymes that catalyzes the dismutation of the superoxide radical (O_2_^−^) into molecular O_2_ and H_2_O_2_ in most prokaryotic cells and in some eukaryotes. Until now, four isoforms of SODs have been identified based on different metal cofactors: Cu/Zn-SOD, Mn-SOD, Fe-SOD and Ni-SOD [5]. Therefore, SODs scavenge superoxide radicals by their dismutation with sequential reduction and oxidation of metal centers—a mechanism known as the ping-pong effect. There are several important drawbacks of the application of natural enzymes as catalysts such as high preparation cost, poor stability under harsh reaction conditions, etc. Therefore, biomimetic catalysts have been developed in order to overcome these limitations. For a biomimetic heterogeneous catalyst to be performant in superoxide scavenging, it is important that the support allows a good interaction with the catalyst and a good accessibility of the substrate to the metal ion active sites. Several metal complexes have proven biomimetic SOD activity when they were immobilized into different supports [6,7,8,9].

Heterogeneous systems are a more convenient alternative to conventional homogeneous catalysts because they have a number of advantages such as easier separation, recovery and handling, and improved stability, as well as overcoming the problem of metal leaching. The heterogenization of homogeneous catalysts has been achieved by their immobilization on different supports such as zeolites [10,11], polymers [12], layered compounds [13], titanium-doped AlPO4-based microporous zeotypes (so-called TAPO materials) [14] and ordered mesoporous carbon [15]. Ordered mesoporous silicas, like MCM-41 and SBA-15, can be used as supports for homogeneous catalysts due to their large surface area that could be easily modified through the surface Si-OH groups. Cu(II) complexes with Schiff bases heterogenized into mesoporous silica have been used for cyclohexene oxidation reactions [16,17,18]. Due to the simultaneous attack of oxidant species both on activated hydrogen at the allylic position and at the double bond, two allylic products (2-cyclohexen-1-ol and 2-cyclohexen-1-one) and two epoxidation products (cyclohexene epoxide and cyclohexane-1,2-diol) can be obtained [19]. Allylic or olefinic oxidations can be facilitated by tuning the reaction parameters, namely the amount of substrate and catalyst, the type of solvent and source of oxygen during oxidation, the oxidant/substrate molar ratio, the time and temperature of the reaction.

Among the metal complexes used as oxidation catalysts, complex combinations of copper have been extensively used for the oxidation of various alkene substrates with different oxidants [20,21,22,23]. A Schiff base has a >C=N-R structure resulting from the condensation of an aldehyde and a primary amine. Therefore, using different aldehydes and amines, a wide variety of Schiff base compounds can be synthesized [24]. Copper(II) Schiff base complexes have been studied for their catalytic properties, being efficient catalysts for oxidation reactions and gaining considerable attention due to their properties. Copper–Schiff base complex catalysts offer advantages in terms of high catalytic activity, versatility and selectivity, as well as simple work-up procedures and low copper content [25]. Their activity can be tuned by changes in ligand types and ligand coordination sites.

However, there are not any reports on heterogeneous biomimetic oxidation catalysts based on Cu(II) complex with the 2-[2-(3-bromopropoxy)benzylideneamino] benzoic acid Schiff base ligand. The presence of two bromine groups in the structure of a complex molecule offers two anchoring sites on the amino-functionalized mesoporous SBA-15 silica support. Therefore, in this paper, we focused on the following goals: (i) to synthesize a new Schiff base ligand and its Cu(II) complex; (ii) to immobilize the complex on an amino-functionalized mesoporous SBA-15 silica support; and (iii) to evaluate the catalytic activity and recyclability of this prepared heterogeneous system for the eco-friendly oxidation of alkenes and as a superoxide radical scavenger.

## 2. Results and Discussion

### 2.1. Characterization of Cu(II) Complex/SBA-15 Catalytic System

**Scheme 1:**
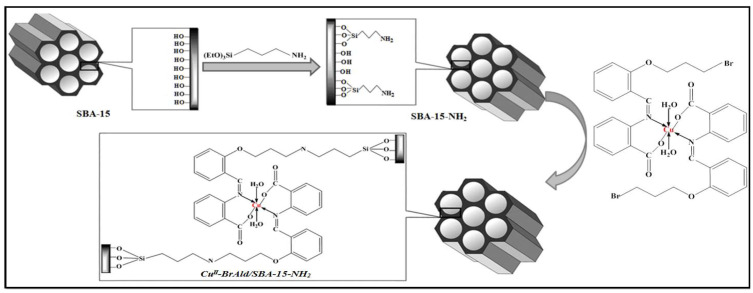
Cu(II)-BrAld/SBA-15-NH2 catalytic system.

A new nanohybrid oxidation catalyst was obtained by covalent grafting a Cu(II) complex with a Schiff base ligand into an amino-functionalized SBA-15 mesoporous silica (Scheme 1). The nanohybrid was synthesized in three steps: (i) post-synthesis functionalization of the mesoporous SBA-15 silica support with 3-aminopropyltriethoxisilane (APTES), (ii) synthesis of 2-[2-(3-bromopropoxy)benzylideneamino] benzoic acid ligand and its Cu(II) complex and (iii) complex grafting onto the amino-functionalized silica support.

#### 2.1.1. Elemental and Thermal Analysis

The elemental analysis of the Cu(II)-BrAld complex (Table 1) showed a Cu/N molar ratio of 1/2, which indicates that Cu(II) has a N_2_O_2_ coordination with the Schiff base ligand and two coordinated H_2_O molecules, as shown in Scheme 1.

**Figure 1:**
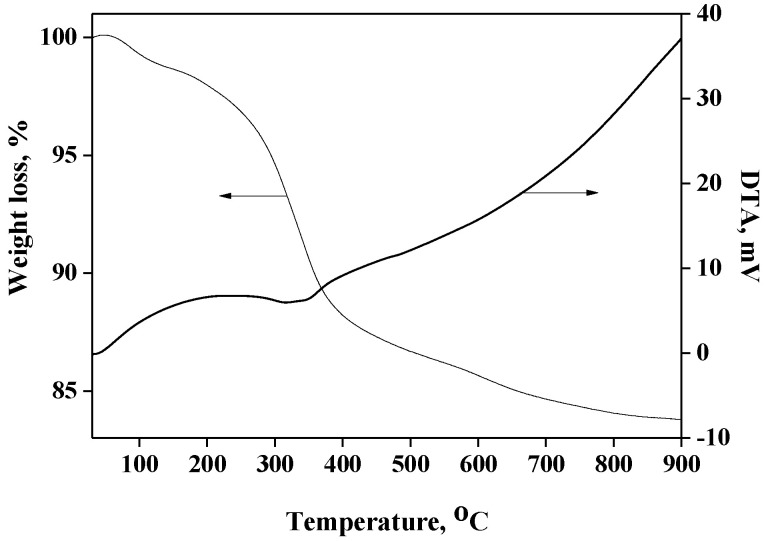
TG-DTA curves for Cu(II)-BrAld/SBA-15-NH_2_.

The TGA profile of Cu(II)-BrAld/SBA-15-NH_2_ is presented in Figure 1. In the first stage, a loss of physically adsorbed water of about 1.5 wt% occurs below 150 °C. Based on the TGA profile, we can consider that the thermal decomposition process of the immobilized complex occurred between 150 and 800 °C (exothermic process), with a weight loss of about 14.5 wt% for the Cu(II)-BrAld/SBA-15-NH_2_ sample. The copper content was determined using AAS after sample dissolution in 10% HF (Table 1). The elemental analysis results (C%, N% and H%) were correlated with the TGA results. The Schiff base ligand loading was 0.18 mmol/g, the copper content 0.09 mmol/g and the complex immobilization yield 75%. After immobilization, the Cu/N molar ratio became 1/4, as the two Br atoms of each complex molecule reacted with two aminopropyl groups of the functionalized support for covalent grafting. The weight loss due to the aminopropyl moiety was 6.43%, corresponding to 1.1 mmol/g.

The disappearance of the C-Br bond in the FTIR spectra of the immobilized complex supports this claim, and the structure presented in Scheme 1 indicates that the homogeneous copper complex remained intact after immobilization into the SBA-15 matrix and the complex geometry did not change during the immobilization process.

#### 2.1.2. Powder X-ray Diffraction

**Figure 2:**
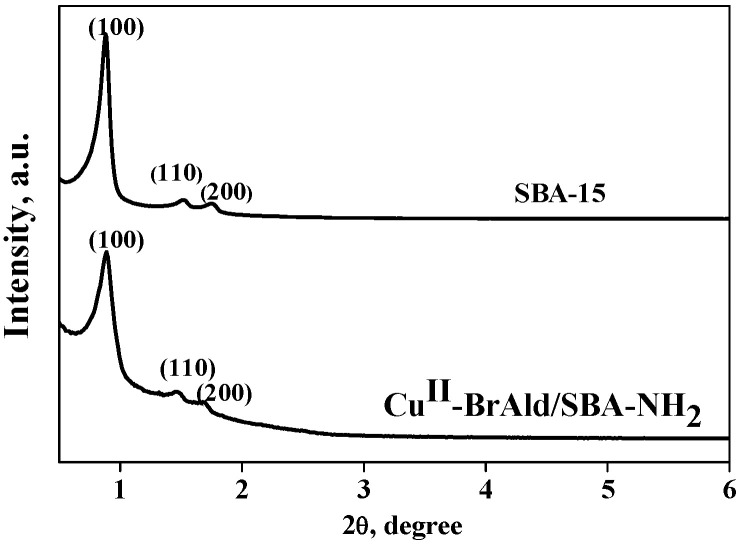
XRD patterns of SBA-15 and Cu(II)-BrAld/SBA-15-NH_2_.

The characteristic mesoporous hexagonal structure of the SBA-15 support and the Cu(II)-BrAld/SBA-15-NH_2_ sample was confirmed by the XRD measurements (Figure 2). The XRD pattern of SBA-15 presented a strong reflection peak at 2θ = 0.89° attributed to the (100) plane and two smaller diffraction peaks at 1.47° for the (110) plane and 1.67° for the (200) plane. After indexation of the three peaks to a hexagonal lattice, the d-spacing values are 99.52, 60.41 and 53.32 Å, respectively, and the unit cell parameter, a_0_, of 115.05 Å, characteristic of a well-ordered SBA-15-type material [26]. However, Cu(II)-BrAld complex immobilization led to minor changes in the low-angle XRD pattern. Thus, an increase in the baseline, a slight shift in the 2θ values of the three characteristic reflections and a decrease in the (100) peak relative intensity for the Cu(II)-BrAld/SBA-15-NH_2_ as compared to the SBA-15 support were evidenced (Figure 2). This behavior can be ascribed to the decrease in the crystallinity of the SBA-15 after functionalization and complex immobilization, as well as to the functionalization in the inner surface of the mesoporous channels, as reported for other functionalized mesoporous silica [27].

#### 2.1.3. N_2_ Adsorption–Desorption Isotherms

**Figure 3:**
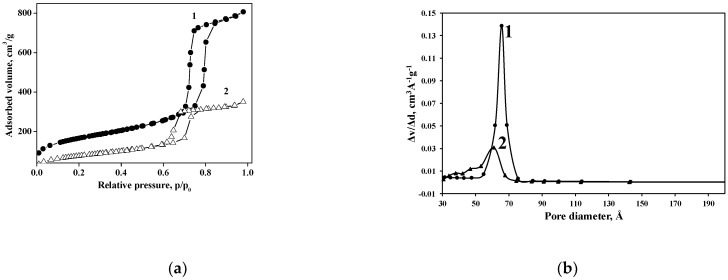
(**a**) N_2_ adsorption–desorption isotherms and (**b**) pore size distribution of SBA-15 (1) and Cu(II)-BrAld/SBA-15-NH_2_ (2).

N_2_ adsorption–desorption isotherm of the Cu(II)-BrAld/SBA-15-NH_2_ sample is an irreversible type IV isotherm, similar to that of pristine SBA-15 (Figure 3). The H1 hysteresis loop, with a sharp desorption step at about 0.70 p/p_0_, is characteristic of well-structured mesoporous materials. The main textural properties of the investigated solids are listed in Table 2.

Changes in N_2_ adsorption–desorption behavior after complex grafting onto an SBA-15 support are mainly determined by the pore blockage and to a lower extent by crystallinity changes. The grafted species were located not only on the outer surface but also inside the mesopores. Consequently, the specific surface area and the volume of mesopores for Cu(II)-BrAld/SBA-15-NH_2_ strongly decreased after Cu(II)-BrAld complex immobilization. 

#### 2.1.4. FTIR Spectroscopy Analysis

**Figure 4:**
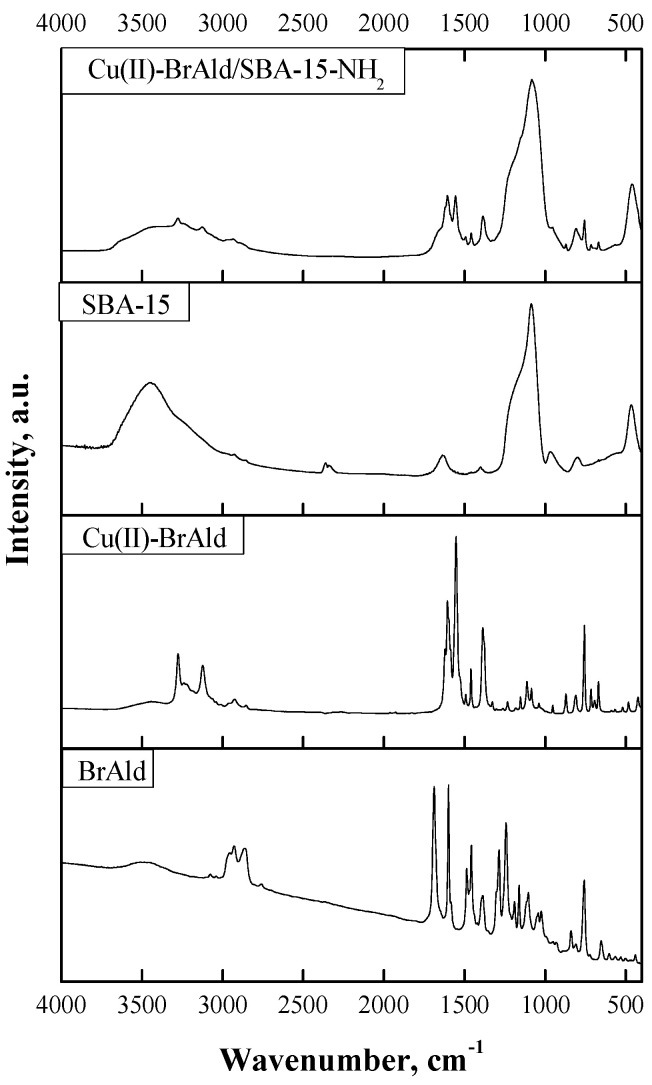
FTIR spectra of BrAld free ligand, Cu(II)-BrAld free complex), SBA-15, Cu(II)-BrAld/SBA-15-NH_2_).

FTIR spectra of the BrAld ligand, Cu(II)-BrAld complex, SBA-15 support and the hybrid Cu(II)-BrAld/SBA-15-NH_2_ illustrated in Figure 4 were recorded in the range of 4000–400 cm*^−^*^1^. The characteristic bands of the Schiff base (BrAld) obtained by the condensation of 2-(3-bromopropoxy) benzaldehyde with 2-aminobenzoic acid are as follows: ν(CH=O) at 1687 cm*^−^*^1^, ν(CH=C) at 1599 cm*^−^*^1^, ν_asim_(COO^−^) at 1490 cm*^−^*^1^, ν_sim_(COO^−^) at 1387 cm*^−^*^1^, ν_asim_(Ar-O-C) at 1243 cm*^−^*^1^, ν_sim_(Ar-O-C) at 1106 cm*^−^*^1^, ν(C-H)aromatic at 758 cm*^−^*^1^ and ν(C-Br) at 652 cm*^−^*^1^. In the Cu(II)-BrAld spectra, the new band at 1608 cm*^−^*^1^ corresponds to the valence vibration of the azomethine (C=N) group, while the band corresponding to the carbonyl group disappeared, proving the Schiff base formation. Furthermore, the bands corresponding to the symmetric and asymmetric vibrations of the Ar-O-C groups were slightly shifted compared with the ligand spectrum, suggesting that the etheric O was not involved in copper coordination. Instead, the carboxylic O was coordinated to the metal ion since the valence vibrations ν_asim_(COO^−^) at 1554 cm*^−^*^1^ and ν_sim_(COO^−^) at 1378 cm*^−^*^1^ were shifted compared with the BrAld ligand. The broad band at ~3450 cm*^−^*^1^ could be assigned to the OH groups from the H_2_O molecules coordinated to the central metallic ion, as the elemental analysis confirmed. The valence vibration of the C-Br bond was at 660 cm*^−^*^1^. In the FTIR spectrum of the free complex, the bands corresponding to the valence vibrations of (Cu-O)carboxyl and Cu-N bonds located at 520 cm*^−^*^1^ and 424 cm*^−^*^1^, respectively, suggest the involvement of carboxylic O and N of the azomethine group in Cu(II) coordination. For SBA-15 silica, there is a large broad band at 3400 cm*^−^*^1^ corresponding to the stretching vibrations of the surface silanol O-H bond and the adsorbed water molecules. The absorption band at 1630 cm*^−^*^1^ could be attributed to deformational vibrations of adsorbed water molecules. For the silica skeleton, the peak centered at 1100 cm*^−^*^1^ corresponds to siloxane, -(Si-O)n-, and that from 970 cm*^−^*^1^ to the Si-O bond stretching. The most obvious changes in the FTIR spectrum of Cu(II)-BrAld/SBA-15-NH_2_ material compared to the free complex and the SBA-15 support are the disappearance of the characteristic band located at 660 cm*^−^*^1^, which corresponds to ν(C-Br), and the appearance of new bands at 2964 cm*^−^*^1^, assigned to the asymmetric stretching of ν(C-H) from the aminopropyl of the sililating agent. Furthermore, the presence of the Cu(II) complex characteristic bands in the spectral range where they do not overlap with the silica backbone (Figure 4) proves the successful immobilization of the complex onto the SBA-15 support by covalent grafting by both Br atoms from the two ligands of the copper complex to the –NH_2_ groups of the functionalized SBA-15 silica.

#### 2.1.5. XPS Analysis

**Figure 5:**
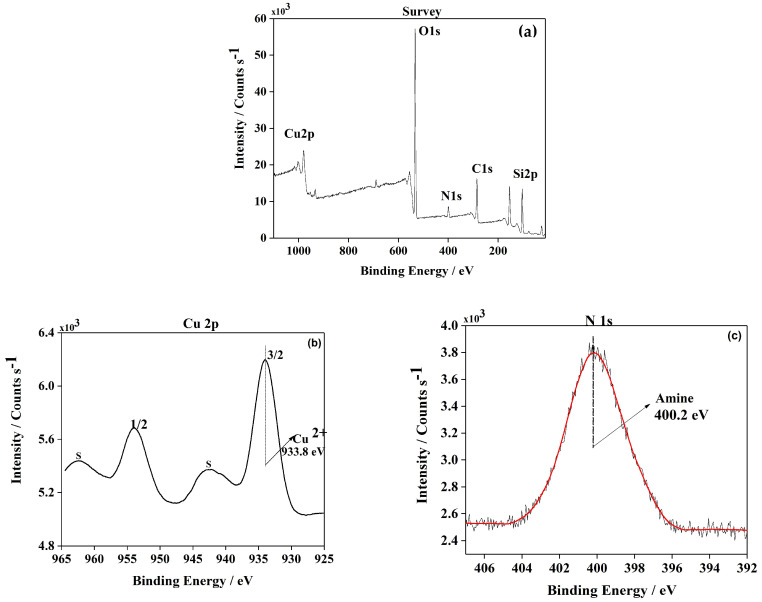
Wide-scan XPS spectrum of (**a**) Cu(II)-BrAld/SBA-15-NH_2_ sample; high-resolution XPS spectra of (**b**) N1s and (**c**) Cu2p_3/2_.

To further characterize this new heterogeneous catalyst, its chemical structure and surface composition were determined by XPS analysis. An XPS spectrum of the Cu(II)-BrAld/SBA-15-NH_2_ sample is shown in Figure 5a. The Si2p peak position for the copper complex immobilized into SBA-15 silica was in agreement with SiO_2_-type material. As expected, the spectra showed peaks for N1s at 400.2 eV assigned to amines and imines (Figure 5b). The spectra concerning Cu2p core-level excitation are presented in Figure 5c. The sample shows two main peaks centered at ~933.8 eV and 964.2 eV associated with Cu 2p_3/2_ and 2p_1/2_, respectively, being accompanied by shake-up satellite peaks. It was proven that satellite bands were generated by an electron transfer from a ligand orbital to the 3d orbital of Cu, confirming that the d level was not filled [28,29,30]. This np(ligand)→3d(metal) transition is characteristic of bivalent copper. The Cu^+^ and Cu^0^ species have filled levels and consequently could not participate in this electron transfer [31]. Furthermore, the Cu2p_3/2_ peak is typical of copper (II) complexes in a mixed N, O coordination sphere [32].

#### 2.1.6. SEM and TEM Microscopy

**Figure 6:**
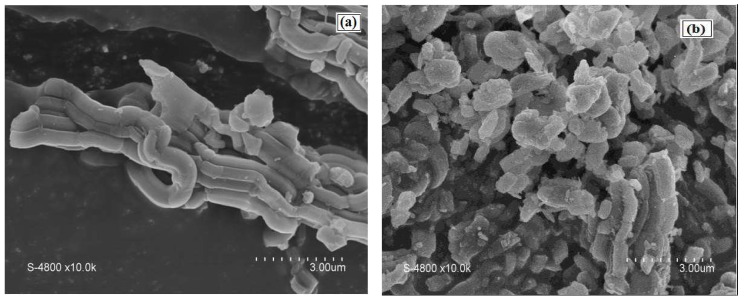
SEM images of (**a**) SBA-15 and (**b**) Cu(II)-BrAld/SBA-15-NH_2_.

**Figure 7:**
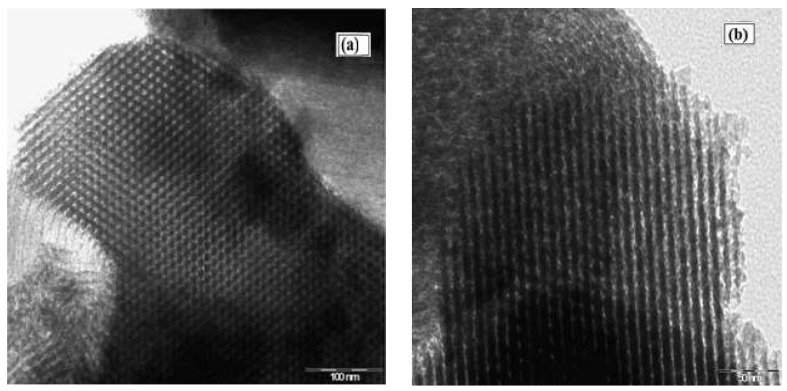
TEM images of (**a**) SBA-15 and (**b**) Cu(II)-BrAld/SBA-15-NH_2_.

An SEM image of the SBA-15 support presented a morphology of aggregate rope-like domains in the macrostructure (Figure 6a). Narrower rope-like domains can be also identified in the SEM image of Cu(II)-BrAld/SBA-15-NH_2_, with a relatively uniform length of 3 μm (Figure 6b). The modification of SBA-15 silica morphology after complex immobilization can be determined by changes in the crystallinity and textural properties. Figure 7 shows some representative TEM images for SBA-15 and Cu(II)-BrAld/SBA-15-NH_2_ materials. For both samples, the characteristic SBA-15 hexagonal mesoporous structure was observed and was in agreement with the XRD characterization.

### 2.2. Catalytic Tests

#### 2.2.1. Catalytic Performances of the Cu(II) Complex/SBA-15

**Scheme 2:**
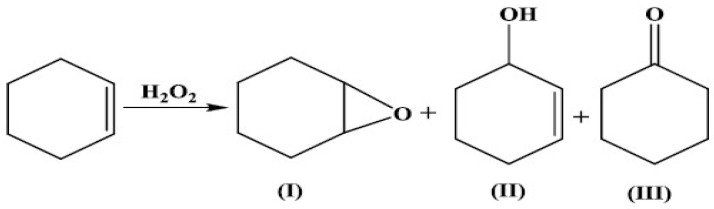
Possible process for cyclohexene oxidation.

The oxidation of cyclohexene with H_2_O_2_ in acetonitrile, under an air atmosphere, was selected as a test reaction to determine the catalytic performances of the SBA-15-supported Cu(II)–Schiff base complex. The optimization of the CH oxidation was previously performed [8] and the best operation parameters were established to be a 5 h reaction run at 60 °C with 0.03 mmol catalyst, 10 mL of solvent and a 2.2/1 H_2_O_2_/C_6_H_10_ molar ratio. Synergic effects for the catalytic activity given by the SBA-15 silica surface and the acetonitrile solvent were observed. In the absence of the catalyst, the reaction did not proceed. Thus, the CH conversion was <1% for the SBA-15 support. Better catalytic performances were obtained for the immobilized complex compared to the free one (Table 3), probably due to the fine distribution of the isolated catalytic sites on the SBA-15 surface. Moreover, the support allows better accessibility of the CH substrate molecules toward the copper ion active sites, improving the energetics of the oxidation reaction compared to free copper complexes. In the optimal conditions, the best catalytic performances were 75% cyclohexene conversion, 45% selectivity for 2-cyclohexen-1-ol and 130 h^−1^ initial turnover frequency. According to GC–MS analyses, the main products of cyclohexene oxidation were cyclohexene oxide (**I**), 2-cyclohexen-1-ol (**II**) and 2-cyclohexen-1-one (**III**), as shown in Scheme 2. These products are formed by the oxidation of both the double bond and the allylic C–H bond. The preponderance of compounds **II** and **III** indicates that the reaction proceeded mainly via the allylic oxidation pathway. Previous studies also evidenced that the immobilization of Cu(II) catalysts may influence the accessibility of the substrates to the active sites, thereby favoring allylic oxidation. In contrast, the free Cu(II) catalyst in solution may exhibit different reactivity due to its interaction with the solvent and the dynamic nature of the reaction environment [33]. These differences in reactivity and selectivity between the immobilized and free Cu(II) catalysts can be attributed to the distinct reaction mechanisms and the influence of the reaction environment. The mechanism of allylic oxidation and epoxidation differs in terms of the intermediates formed during the reaction. The allylic oxidation process is initiated by the abstraction of a hydrogen atom from the allylic position, followed by the formation of an allylic radical. The radical then reacts with oxygen to form a peroxy radical, which can undergo further reactions to form the desired product. On the other hand, epoxidation involves the formation of a metal–oxygen intermediate, which then reacts with the alkene to form the epoxide. The intermediate is formed by the reaction of the metal catalyst with a peroxyacid or other oxidizing agent. The intermediate then reacts with the alkene to form the epoxide. The difference in the reaction mechanism leads to different selectivities for the two reactions. The immobilization of the Cu(II) catalyst may influence the accessibility of the substrates to the active sites, thereby favoring the allylic oxidation [34].

These results are in agreement with previously reported results concerning salicylaldimine complexes of Cu(II) and Co(II) immobilized on silica supports (MCM-41, SBA-15 and Davisil 710) as catalysts for cyclohexene oxidation using hydrogen peroxide as an oxidant under an oxygen atmosphere [18]. It was observed that the copper catalysts generally produced almost equal amounts of alcohol and ketone and that SBA-15 led to higher levels of alcohol compared to MCM-41, which tends to favor ketone. 

When other supports were used as hosts for the Cu(II)–Schiff base complexes, superior activity toward a CH oxidation reaction and higher selectivity to epoxide or ketone were obtained [35,36].

The CH oxidation was accompanied by the self-decomposition of H_2_O_2_ as a side reaction. The effective utilization of H_2_O_2_ was 61% for the Cu(II)-BrAld/SBA-15-NH_2_ catalyst, indicating the participation of the oxidizing agent in other inefficient secondary reactions besides the tested oxidation reaction.

By comparing the CH conversion and selectivity for allylic oxidation compounds with other heterogeneous copper complexes, this new catalyst presents a moderate conversion and poor selectivity, probably due to a spatial environment of the metal center that did not allow an optimal interaction with the substrate molecules. When a Cu(Salen) complex intercalated with α-zirconium phosphate was tested for the oxidation of CH using tert-butylhydroxide as an oxidant, under optimized conditions, the maximum conversion was 26.71% with cyclohexanone as the major product (49.80%) [37]. CH oxidation with H_2_O_2_ was studied with a catalyst based on M41S-type mesoporous silicate spherical particles containing copper nanospecies with a maximum conversion of 30% and allylic oxidation as the main reaction [38]. Heterogeneous Cu(II) complexes with unsubstituted and tertiary-butyl-substituted salycilaldimine ligands were immobilized on silica supports and tested for cyclohexene oxidation using hydrogen peroxide as an oxidant under an oxygen atmosphere [16]. The maximum CH conversion was 84%, and 2-cyclohexen-1-ol and 2-cyclohexen-1-one prevailed as allylic oxidation products.

#### 2.2.2. Stability of Cu(II) Complex/SBA-15 in the Cyclohexene Oxidation Reaction

The reusability of this new hybrid Cu(II)-BrAld/SBA-15-NH_2_ heterogeneous catalyst was further investigated by the measurement of its catalytic performances over five cycles of CH oxidation. The catalyst was stable during the test, with a slight decrease in the initial TOF value in the fifth run (Table 4). After each cycle, the recovered catalyst had almost the same copper content as the fresh catalyst (0.55% Cu), according to the AAS measurements. This further indicates no leaching of the metal from the SBA-15-hosted copper complex and its stability. Covalent grafting onto the mesoporous silica surface stabilized the catalytically active copper complex and the oxidizing medium did not affect the surrounding of the catalytic active sites. By using SBA-15 silica characterized by larger pores than other mesoporous or microporous supports, the substrate diffusion during the repeated cyclohexene oxidation cycles was not impeded and the adsorption of the reaction products on the support was partially eliminated. Both CH conversion and TOF values were, however, slightly diminished after repeated use, probably due to a change in the morphology of the mesoporous material or in the catalytically active center surroundings.

### 2.3. Superoxide Dismutase (SOD) Activity of Cu(II)-BrAld/SBA-15-NH_2_ Catalyst

The biomimetic comportment of free or immobilized Cu(II)-BrAld complexes was tested for SOD activity [8]. All materials, except the silica support, presented catalytic activity in the dismutation reaction of superoxide radicals (O_2_^−^). The concentration for 50% inhibition of the NBT reduction was 33 µM for the free Cu(II)-BrAld complex. For the Cu(II)-BrAld/SBA-15-NH_2_ heterogeneous catalyst, the SOD activity changed after Cu(II) complex immobilization and the 50% inhibition concentration value was 24 μM. When compared with other copper complexes immobilized into different supports and used as biomimetic catalysts, the SOD activity of this new catalyst is comparable with and even better [6,7,8,39,40,41]. A possible explanation for the increased SOD activities of the immobilized complexes is that the silica matrix probably provides an optimal environment for the complex that mimics the active site of the natural SOD enzyme, allowing better dispersion of the metallic sites and accessibility of the substrate to these sites. Furthermore, the heterogeneous catalyst could be more easily recovered and reused compared with the free complex.

## 3. Materials and Methods

### 3.1. Materials and Equipment

Poly(ethyleneglycol)-block-poly(propyleneglycol)-block-poly(ethyleneglycol) (Pluronic P123, Mav = 5800), tetraethylorthosilicate (TEOS, 98%) and 3-aminopropyltriethoxisilane (APTES, 99%) (BASF, Ludwigshafen, Germany and Sigma-Aldrich, St. Louis, MO, USA) were used without further purification for amino-functionalized mesoporous silica support synthesis. CuCl_2_∙2H_2_O, NH_4_OH, 2-aminobenzoic acid, benzaldehyde and 1,3-dibromopropane (Sigma-Aldrich, St. Louis, MO, USA) were used for the catalyst synthesis. The catalytic tests were performed using cyclohexene (Aldrich) as an oxidation substrate and H_2_O_2_ (Merck, Darmstadt, Germany) as an oxidant. Riboflavin, L-methionine and nitro blue tetrazolium (NBT) reagents were used in biochemical tests of superoxide dismutase activity. In this study, methanol, ethanol, dimethylformamide (DMF), toluene, diethyl ether, dichloromethane and acetonitrile (Sigma-Aldrich, St. Louis, MO, USA) were used as solvents. A Bruker AXS D8 diffractometer (Karlsruhe, Germany) was used for powder X-ray diffraction (XRD) measurements in a 2θ low-angle range from 10 to 60 with Cu Kα radiation. A Micromeritics ASAP 2010 (Norcross, GA, USA) instrument was used to record the N_2_ adsorption–desorption isotherms at −196 °C after vacuum-degassing the samples at 500 °C for 12 h. From the isotherms, the specific surface area was calculated using the BET method and the mesopore volume was determined at the end of capillary condensation. The BJH method and the Harkins–Jura standard isotherm for the desorption branch of the isotherms were used to obtain the distribution of pore size. The FTIR spectra were recorded using a Bruker Alpha spectrometer (Rosenheim, Germany). For the XPS measurements, a SPECS PHOIBOS 150 MCD instrument (Berlin, Germany) with monochromatic Al Kα radiation (1486.69 eV) was used at 14 kV and 20 mA and a pressure lower than 10^−9^ mbar. The binding energy scale was charge-referenced to the C 1s photoelectron peak at 285 eV. Spectra deconvolution was accomplished with Casa XPS (Casa Software Ltd., Devon, UK). The copper content was determined by flame atomic absorption spectrometry (AAS) on a Spectra AA-220 Varian Spectrometer (Victoria, Australia) with an air acetylene flame. An elemental analysis apparatus, Fisons EA1108 (Thermo Fisher, Waltham, MA, USA), performed the elemental analysis (C, H and N). A Netzsch TG 209C (UK) thermobalance in nitrogen flow was used for the thermogravimetric analysis (TG/DTA).

### 3.2. Synthesis Procedures

#### 3.2.1. Preparation of Functionalized SBA-15 Silica

SBA-15 silica support was synthesized according to a literature procedure [42] as follows: 1.5 g of Pluronic P123 surfactant templating agent was dispersed in a mixture of 15 g of H_2_O and 45 g of 2 M HCl by stirring for 4 h at 40 °C. A 3.15 g quantity of TEOS was further added and the reaction mixture was maintained under stirring at 40 °C for 24 h. After a hydrothermal treatment in a Teflon-lined autoclave at 100 °C for 2 days, the solid was centrifuged, filtered, washed with deionized water and dried in air at room temperature. The final SBA-15 support was obtained after solid calcination at 550 °C for 8 h under airflow. The amino-functionalization of the inorganic support was carried out according to a post-grafting procedure [43] consisting of mixing 50 mL of dry toluene with 1 g of activated SBA-15 silica and 1 mL of APTES, followed by stirring the solution for 4 h at the reflux temperature of toluene. Thereafter, the NH_2_-functionalized silica was filtered and washed (using toluene, ethylic alcohol and diethyl ether successively). The surfactant was removed by a Soxhlet extraction with diethyl ether/dichloromethane (*v*/*v*, 1/1) at 100 °C. The solid was dried at 130 °C overnight (referred to as SBA-15-NH_2_).

#### 3.2.2. Synthesis of the Cu(II) Complex

The 2-(3-bromopropoxy)benzaldehyde ligand was synthesized as described in the literature [44] using benzaldehyde and 1,3 dibromopropane and was denoted as BrAld. For the Cu(II) complex synthesis, the suspension of 2-aminobenzoic acid (2.048 mol) in absolute ethanol (10 mL) was added to a solution of 2-(3-bromopropoxy) benzaldehyde (2.05 mol) in absolute ethanol (10 mL) and was kept under continuous stirring at RT for 2h. Subsequently, 10 mL of an ethanolic solution of CuCl_2_∙2H_2_O (1.02 mol) was added to the mixture and its pH was adjusted to 8 with a NH_4_OH solution; the as-obtained mixture was refluxed for one hour. The obtained green precipitate was filtered, washed with water (2 × 10 mL), ethanol (2 × 10 mL) and diethyl ether (2 × 10 mL) and dried at room temperature for 24 h (referred to as Cu(II)-BrAld). 

#### 3.2.3. Cu(II) Complex/SBA-15 Catalyst

The complex Cu(II)-BrAld (0.1 g) and 0.5 g of activated SBA-15-NH_2_ (3 h at 120 °C under high vacuum) were reacted for 48 h in 10 mL DMF at reflux under continuous stirring and in a N_2_ atmosphere (Scheme 1). The resulting heterogeneous catalyst (referred to as Cu(II)-BrAld/SBA-15-NH_2_) was filtered out and washed with toluene (2 × 30 mL), ethanol (2 × 30 mL) and diethyl ether (2 × 30 mL). The final product was dried at 110 °C under a vacuum for 4 h.

### 3.3. Catalytic Oxidation of Cyclohexene

The oxidation of cyclohexene (CH) with H_2_O_2_ was used as a test reaction for the measurement of Cu(II)-BrAld/SBA-15-NH_2_ activity, as previously described [8]. A total of 2.26 mmol of CH, 0.03 mmol of catalyst and 10 mL of acetonitrile were added successively in a temperature-controlled two-necked round-bottom flask with a reflux condenser. When the temperature reached 60 °C, hydrogen peroxide (30% H_2_O_2_) was added dropwise. The products were analyzed using a Thermo DSQ II system (Waltham, MA, USA) with a GC-Focus gas chromatograph, a DSQ II mass spectrometer and a Thermo TR-5MS 30 m × 0.25 ID × 0.25 μm film capillary column [8]. 

The conversion, selectivity and turnover frequency (TOF) were calculated as follows:

Conversion (%) = 100 × (C_0_ − C_t_)/C_0_ (1)

Product selectivity (%) = [(product formed (mol)/total product detected (mol)] × 100(2)

TOF = Substrate converted (mol)/[Copper in catalyst (mol) × reaction time (h)](3)

where C_0_ = initial concentration (mol); C_t_ = final concentration (mol).

H_2_O_2_ consumption was determined by an iodometric titration and H_2_O_2_ efficiency was calculated as the percentage of this reagent converted to oxidized products. 

The reusability of the catalyst was tested for five consecutive runs of CH oxidation.

### 3.4. Catalysis of Superoxide Dismutation

In order to assess the SOD activity of the free or immobilized Cu(II) complex, as well as that of the SBA-15 support, the quantity of enzyme inhibiting the NBT photoreduction by 50% (in μM) was determined for each sample, using the Beachamp–Fridovich reaction [45] as we described elsewhere [8,9].

## 4. Conclusions

A novel catalyst based on a Cu(II) complex with 2-[2-(3-bromopropoxy)benzylideneamino] benzoic acid covalently grafted on an amino-functionalized SBA-15 support was prepared and tested in the oxidation process of cyclohexene with 30% H_2_O_2_ or as a scavenger of superoxide radical anions. Furthermore, this catalyst can be reused in five cycles of cyclohexene oxidation without loss of its catalytic activity and selectivity. However, even though the catalytic performances of the heterogenized Cu(II) complexes on the SBA-15 support were better in comparison with their homogeneous analogs, they were moderate when compared with other similar heterogeneous catalysts. The obtained results are encouraging for the design of new heterogeneous catalysts by the immobilization of metal complexes into mesoporous silica supports that could be used either for efficient alkene oxidation or as artificial enzymes with superoxide scavenging activity.

## Figures and Tables

**Table 1:** Analytical data of Cu(II)–Schiff base complex free or SBA-15-immobilised.

Compound	Analytical Data ^a^ (%)				Complex Amount ^b^ (mg g^−1^)	Cu Amount (mg g^−1^)	Cu/N Molar Ratio
	C	H	N	Cu			
Cu(II)-BrAld, C_34_H_34_O_8_N_2_Br_2_Cu	49.54 (49.66)	4.15 (4.14)	3.40 (3.41)	7.52 (7.73)	-		1/2
Cu(II)-BrAld/SBA-15-NH_2_	7.92 (7.84)	0.69 (0.65)	0.51 (0.53)	0.56 (0.57)	119.07	5.72	1/4

^a^ Calculated values are shown in parentheses; for the immobilized complex, the C%, H% and N% were calculated only for the ligand amount corresponding to the Cu% determined by AAS, considering a metal-to-ligand ratio of 1/2 and subtracting the aminopropyl group previously grafted. ^b^ Determined by TGA after subtraction of the amino propyl moiety previously grafted on the silica support.

**Table 2:** Textural properties of calcined and modified SBA-15.

Sample	a_0_ (nm) ^a^	S_BET_ (m^2^g^−1^) ^b^	V_p _ (mL g^−1^) ^c^	D_p_ (nm) ^d^	Wall Thickness (nm) ^e^
SBA-15	11.3	600	1.1	7.8	3.5
Cu(II)-BrAld/SBA-15-NH_2_	11.2	287	0.46	6	5.2

^a^ a_0_ = 2d_100_/$\sqrt{3}$; ^b^ S_BET_—specific surface area; ^c^ V_p_—mesopore volume; ^d^ D_p_ = pore diameter; ^e^ Wall thickness = a_0_–D.

**Table 3:** Catalytic performances of the Cu(II) complex.

Sample	CH Conversion (%)	TOF (h^−1^)	Selectivity (%)		
			I	II	III
Cu(II)-BrAld	11	12	68	22	10
Cu(II)-BrAld/SBA-15-NH_2_	75	130	15	45	40

Reaction conditions: catalyst (0.03 mmol), substrate (2.26 mmol), ACN (10 mL), H_2_O_2_ (4.75 mmol), 5 h, 60 °C. Formed products: cyclohexene oxide (**I**), 2-cyclohexen-1-ol (**II**) and 2-cyclohexen-1-one (**III**).

**Table 4:** Reusability of Cu(II)-BrAld/SBA-15-NH_2_ catalyst.

No. of Cycle	CH Conversion %	Initial TOF (h^−1^)
1	75	130
2	74	129
3	73	127
4	72	126
5	70	125

## Data Availability

Data is contained within the article.
